# Proposal for a structured computed tomography report in the evaluation of pancreatic neoplasms based on expert opinions

**DOI:** 10.1590/0100-3984.2016.0211

**Published:** 2018

**Authors:** Paulo Gustavo Maciel Lopes, Carlos Alberto Matsumoto, Edson José Lobo, Giuseppe D'Ippolito

**Affiliations:** 1 MD, Radiologist in the Department of Diagnostic Imaging of the Escola Paulista de Medicina da Universidade Federal de São Paulo (EPM-Unifesp), for the Laboratório DASA, and for the Laboratório CDB, São Paulo, SP, Brazil.; 2 MD, Radiologist in the Department of Diagnostic Imaging of the Escola Paulista de Medicina da Universidade Federal de São Paulo (EPM-Unifesp) and for Fleury Medicina Diagnóstica, São Paulo, SP, Brazil.; 3 Associate Professor in the Department of Surgery of the Escola Paulista de Medicina da Universidade Federal de São Paulo (EPM-Unifesp), SP, Brazil.; 4 Tenured Adjunct Professor in the Department of Diagnostic Imaging of the Escola Paulista de Medicina da Universidade Federal de São Paulo (EPM-Unifesp), São Paulo, SP, Brazil.

**Keywords:** Pancreatic neoplasms/diagnostic imaging, Tomography, X-ray computed, Neoplasm staging, Consensus, Neoplasias pancreáticas/diagnóstico por imagem, Tomografia computadorizada por raios X, Estadiamento de neoplasias, Consenso

## Abstract

**Objective:**

To create a structured computed tomography (CT) report for the systematic evaluation of pancreatic ductal adenocarcinoma (PDAC), based on the opinions of clinicians and surgeons.

**Materials and Methods:**

This was a prospective study in which we applied a 21-item questionnaire to experts in pancreatic diseases in order to create a model of a structured abdominal CT report. The questionnaire addressed the location and size of PDACs, as well as their effects on adjacent structures and on the vasculature, together with metastases. We used a Likert scale to determine which of those parameters should be included in the model.

**Results:**

A total of 18 experts (12 surgeons and 6 clinicians) from 9 institutions completed the questionnaire. All of the experts agreed that the following (if present) should be described in the CT report on a PDAC: the degree of enhancement; the diameter and location of the lesion; pancreatic duct obstruction; biliary dilatation; pancreatic atrophy; liver metastases; peritoneal nodules; ascites; lymph node enlargement; and invasion of adjacent structures. More than 80% of the experts agreed that the report should also describe the relationship between the PDAC and the surrounding vasculature.

**Conclusion:**

We have developed a template for a CT report on patients with PDAC, based on the opinions of experts involved in the treatment of such patients.

## Abstract (pt)

**Objetivo:**

Criar um modelo de laudo estruturado de tomografia computadorizada (TC) para a avaliação sistemática do adenocarcinoma ductal de pâncreas (ADP) com base na opinião de clínicos e cirurgiões.

**Materiais e Métodos:**

Realizamos estudo prospectivo aplicando um questionário abrangendo 21 tópicos para um modelo de laudo estruturado de TC do abdome na avaliação do ADP, contemplando dados sobre localização e tamanho tumoral, efeito sobre estruturas adjacentes, comprometimento vascular e presença de metástases. Utilizamos a escala de Likert para definir a pertinência de se inserir uma determinada informação.

**Resultados:**

Obtivemos respostas de 18 especialistas de 9 instituições (12 cirurgiões e 6 clínicos). Todos concordaram que deveriam ser descritos o grau de realce, o diâmetro da lesão, a localização e a presença ou a ausência de: obstrução do ducto pancreático, dilatação biliar, atrofia pancreática, metástases hepáticas, nódulos peritoniais, ascite, linfonodomegalias e invasão de estruturas adjacentes. Mais de 80% concordaram que deveria ser mencionada a relação do tumor com os vasos circunjacentes.

**Conclusão:**

Elaboramos um modelo de laudo estruturado de TC para a avaliação do ADP de pâncreas, atendendo às expectativas dos especialistas envolvidos no atendimento destes pacientes.

## INTRODUCTION

Pancreatic ductal adenocarcinoma (PDAC) is the fourth leading cause of cancer death in the United States^(1)^. In Brazil, it accounts for approximately 2% of all malignancies and 4% of all cancer deaths^(2)^.

Although surgery provides the possibility of cure for PDAC^(3)^, curative or radical surgery is possible in only a small fraction of patients with the disease. When a PDAC is located in the pancreatic head, it is resectable in 15-20% of cases, compared with only 10% when it is located in the body or tail^(4)^. Although many consider PDAC to be incurable, radical surgery offers a five-year survival rate of approximately 20%, compared with an overall rate of less than 5%^(5)^ . In addition, survival is longer and quality of life is better among patients who undergo resection than among those who undergo other, nonradical, forms of treatment^(5)^.

The indication of surgical treatment for the purpose of resection of a PDAC depends on criteria related to the clinical conditions of the patient and stage of the disease^(6)^. Among the surgical options, the surgical procedure most often performed in patients with PDAC of the pancreatic head is pancreaticoduodenectomy, which is associated with morbidity and mortality rates of up to 20% and 2%, respectively, even at facilities where a high volume of surgical procedures are performed^(6)^. Accurate preoperative evaluation and locoregional staging of PDACs, through imaging methods, can preclude the need for curative surgery and facilitate surgical planning, thus reducing the rate of complications inherent to the procedure^(7,8)^. In the absence of distant metastases (to the liver, lungs, peritoneum, etc.), the focus falls mainly on the involvement of blood vessels surrounding the pancreas, primarily the superior mesenteric vein, superior mesenteric artery, portal vein, common hepatic artery, and celiac trunk. On the basis of such analyses, PDACs are then classified as resectable, unresectable, or indeterminate, the last also being referred to as threshold or borderline PDACs^(9)^.

Magnetic resonance imaging and, more often, computed tomography (CT) are the diagnostic imaging tools routinely used in the staging and therapeutic planning of cases of pancreatic disease^(7,8,10,11)^. For patients with PDAC, a multidisciplinary evaluation involving surgeons, oncologists, and radiologists, which is now routine, prevents many inappropriate practices. Those joint analyses are indispensable to the preoperative and postoperative evaluation, as well as the response to treatment, improving patient care and encouraging better performance by the different groups of professionals, who can thus offer therapeutic approaches consistent with the degree of involvement and the extent of the disease^(12)^. The best results are obtained when the radiology report is clear, detailed, and well structured, containing all of the information necessary to devise the most appropriate treatment strategy for each patient^(13)^.

Recently, several groups of specialists have suggested adopting structured imaging reports for various clinical situations, including aortic aneurysm^(14)^, rectal cancer^(15)^, prostate cancer^(16)^, head/neck cancer^(17)^, and pancreatic adenocarcinoma^(18,19)^. Although this model of structured reporting is desired by many specialists and could improve cancer patient care, it is still little used in the field of radiology^(20)^.

The objective of the present study was to construct a model of a structured abdominal CT report. The model was based on the opinion of specialists and designed to meet the needs inherent to therapeutic planning in patients with PDAC.

## MATERIALS AND METHODS

This was a prospective, descriptive, cross-sectional study. The study was approved by the Research Ethics Committee of the Federal University of São Paulo School of Medicine, in the city of São Paulo, Brazil. Initially, we organized a meeting of surgeons and gastroenterologists, all of whom were specialists in pancreatic diseases and members of the Pancreatic Diseases Study Group, which is a multidisciplinary medical community that holds monthly gatherings of professionals from various educational institutions, aimed at continuing education, as well as the exchange of knowledge and experiences related to diseases of the pancreas. At the meeting, we presented a project designed to establish a model of a CT report for patients with suspected PDAC, based on data in the literature^(17)^. To that end, we asked the attendees to complete a questionnaire that covered 21 imaging aspects of a PDAC, in order to determine which information should be contained in an abdominal CT report for a patient with pancreatic cancer (Table 1). None of the participants were offered any incentive.

**Table 1:** Aspects that should be included in a radiology report designed for use in patients with pancreatic neoplasms

**Morphological evaluation of the pancreatic lesion**
Degree of lesion enhancement: hypovascular, isovascular, or hypervas-cular?
Size of the lesion at its greatest diameter? (when measurable)
Location of the lesion in the pancreas: uncinate process, head, body, or tail?
Abrupt narrowing or obstruction of the pancreatic duct (with or without upstream dilatation or parenchymal atrophy)?
Obstruction of hepatobiliary duct (with or without upstream dilatation)?
**Evaluation of arterial involvement**
Superior mesenteric artery, celiac trunk, and common hepatic artery: affected or unaffected?
Degree of contact between the tumor and the artery: ≤ or > 180º?
Focal stenosis or irregular vessel contour?
Involvement of the common hepatic artery extending to its bifurcation or major branches?
Arterial anatomic variation: presence/absence, unaffected/affected, degree of contact between the tumor and the artery, focal stenosis, or irregular vessel contour?
**Evaluation of venous involvement**
Portal trunk and superior mesenteric vein: unaffected, affected, or com-pletely occluded?
Degree of contact between the tumor and the vein: ≤ or > 180º?
Focal stenosis or irregular vessel contour: present or absent?
Extension to the first branch of the superior mesenteric vein: present or absent?
Venous thrombosis in the portal vein, superior mesenteric vein, or splenic vein: present or absent?
Collateral circulation (peripancreatic, mesenteric, in the hepatic hilum, or in the left hypochondrium): present or absent?
**Evaluation of extrapancreatic involvement**
Hepatic lesions (suspicious, indeterminate or benign): present or ab-sent?
Peritoneal or omental nodules: present or absent?
Ascites: present or absent?
Suspicious or enlarged hepatic hilar, celiac trunk, splenic, periaortic, or interaortocaval lymph nodes: present or absent?
Invasion of adjacent structures: present or absent?

Using a Likert scale^(21)^, the participants scored the various aspects on whether or not they should be included in the report, responding to each statement, as follows: 1 = totally disagree; 2 = partially disagree; 3 = no opinion; 4 = partially agree; or 5 = totally agree. To perform the statistical analysis, scores of 1 and 2 were grouped, because they both indicated disagreement with the need to include a certain item in the structured report. Likewise, scores of 4 and 5 were grouped, because they both indicated agreement with the need to include a certain item. Scores of 3, indicating indifference to the inclusion of a given item, were analyzed separately. In addition, for each analyzed item, a mean was calculated from the answers of all study participants, ranging from 1 to 5, the means closer to 5 and closer to 1 indicating greater agreement and disagreement, respectively, with the inclusion of a given item in the structured report. To analyze the information collected, we used descriptive statistics, calculating means and frequencies.

## RESULTS

The questionnaire was completed by 18 specialists from 9 educational institutions in the city of São Paulo. Of those 18 specialists, 12 (66.7%) had more than five years of experience in their professional activity. Twelve of the participants (66.7%) were surgeons and 6 (33.3%) were clinicians.

**Figure 1:**
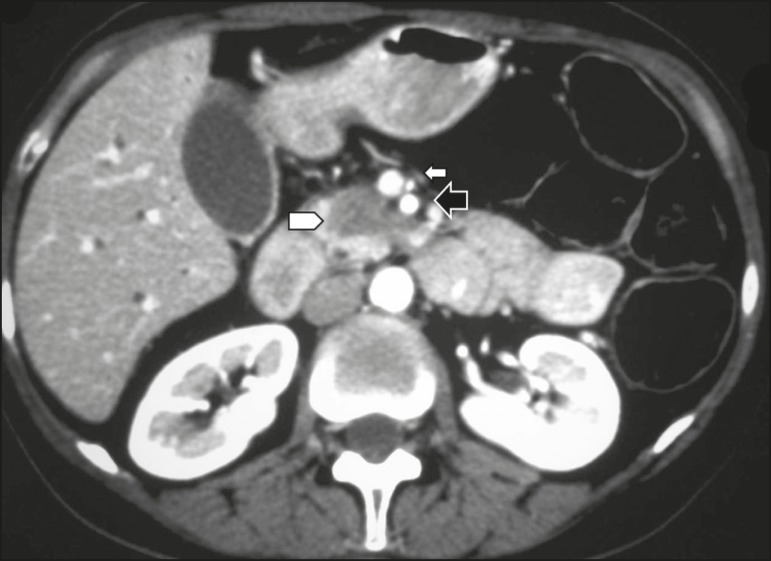
Neoplasm in the pancreatic head, with no signs of vascular invasion. Hypovascular mass in the pancreatic head (arrowhead). Superior mesenteric artery (black arrow) and superior mesenteric vein (small white arrow). Note that the degree of contact between the tumor and those vessels was < 180°, without thrombosis or parietal distortion, indicating that was no vascular invasion.

For all of the aspects analyzed, more than 83% of the participants agreed with their inclusion in the radiology report, the mean score ranging from 4.44 to 5 (Table 2). Figures 1 through 4 illustrate examples of cases of PDAC and its effects on adjacent structures presented by the group of specialists.

**Table 2:** Distribution of agreement among the study participants regarding the inclusion of the various CT aspects of a PDAC.

Aspects	Mean	Agreement
**Morphological evaluation of the pancreatic lesion**		
Degree of enhancement: hypovascular, isovascular, or hypervascular?	5.00	100.00%
Size of the lesion at its greatest diameter? (when measurable)	4.94	100.00%
Location of the lesion in the pancreas: uncinate process, head, body, or tail?	4.94	100.00%
Abrupt narrowing or obstruction of the pancreatic duct (with or without upstream dilatation or parenchymal atrophy)?	5.00	100.00%
Obstruction of hepatobiliary duct (with or without upstream dilatation)?	5.00	100.00%
**Evaluation of arterial involvement**		
Superior mesenteric artery, celiac trunk, and common hepatic artery: affected or unaffected?	4.88	94.12%
Degree of contact between the tumor and the artery: ≤ or > 180º?	4.44	83.33%
Focal stenosis or irregular vessel contour?	4.67	88.89%
Involvement of the common hepatic artery extending to its bifurcation or major branches?	5.00	94.44%
Arterial anatomic variation: presence/absence, unaffected/affected, degree of contact between the tumor and the artery, focal stenosis, or irregular vessel contour?	5.00	100.00%
**Evaluation of venous involvement**		
Portal trunk and superior mesenteric vein: unaffected, affected, or completely occluded?	5.00	100.00%
Degree of contact between the tumor and the vein: ≤ or > 180º?	4.78	94.44%
Focal stenosis or irregular vessel contour: present or absent?	4.82	100.00%
Extension to the first branch of the superior mesenteric vein: present or absent?	4.67	88.89%
Venous thrombosis in the portal vein, superior mesenteric vein, or splenic vein: present or absent?	5.00	100.00%
Collateral circulation (peripancreatic, mesenteric, in the hepatic hilum, or in the left hypochondrium): present or absent?	4.83	100.00%
**Evaluation of extrapancreatic involvement**		
Hepatic lesions (suspicious, indeterminate or benign): present or absent?	4.89	100.00%
Peritoneal or omental nodules: present or absent?	4.94	100.00%
Ascites: present or absent?	4.94	100.00%
Suspicious or enlarged hepatic hilar, celiac trunk, splenic, periaortic, or interaortocaval lymph nodes: present or absent?	4.94	100.00%
Invasion of adjacent structures: present or absent?	5.00	100.00%

**Figure 4:**
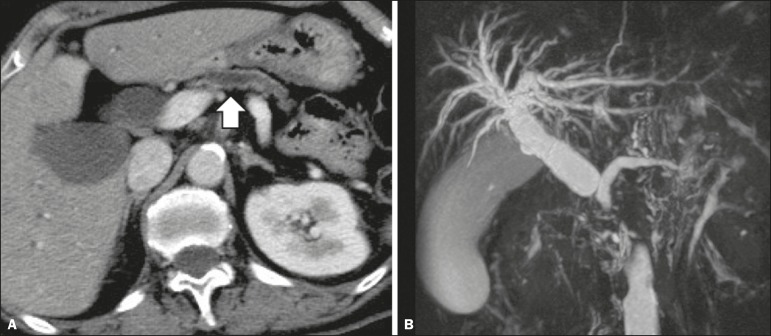
Contrast-enhanced CT (**A**) and magnetic resonance cholangiography (**B**). Neoplasm of the pancreatic head, causing dilatation of the main pancreatic duct (arrow in **A**), with atrophy of the body and tail of the pancreas, together with dilatation of the biliary tract.

### Evaluation of the morphology of pancreatic lesions

All participants agreed that certain aspects should be included in the radiology report. Those aspects were the degree of lesion enhancement (hypovascular, isovascular, or hypervascular); the size of the lesion at its greatest diameter (when measurable); the location of the lesion; narrowing or obstruction of the main pancreatic duct, with or without upstream dilatation and with or without parenchymal atrophy; and obstruction of the hepatobiliary duct, with or without upstream dilatation.

### Evaluation of arterial involvement

The question regarding whether involvement (or lack thereof) of the superior mesenteric artery, celiac trunk, and common hepatic artery should be included in the radiology report was answered by 17 of the 18 participants. Of those 17 respondents, 16 (94%) agreed that arterial involvement should be described in the report and 1 (6%) had no opinion. As for the degree of contact between the tumor and the surrounding arteries (≤ or > 180°), 15 (83%) of the 18 respondents agreed that it should be described in the report, 2 (11%) had no opinion, and 1 (6%) disagreed completely. Regarding the presence or absence of focal stenosis or irregular vessel contour, 16 (89%) of the 18 respondents agreed that it should be described in the report and 2 (11%) had no opinion. Regarding whether involvement of the common hepatic artery extended to its bifurcation or major branches, 17 (94%) agreed with the inclusion of that information and 1 (6%) had no opinion. All participants agreed that the presence or absence of arterial anatomic variations should be included in the report, as should whether or not those variations are affected, the degree of contact between the tumor and the variations, and the presence of focal stenosis or irregular vessel contour.

### Evaluation of venous involvement

All participants agreed that the radiology report should describe the status of the trunks of the portal vein and superior mesenteric vein, in terms of their involvement and degree of occlusion, as well as whether or not there is venous thrombosis or collateral circulation. As for the degree of contact between the tumor and the surrounding veins (≤ or > 180°), 17 (94%) of the 18 respondents agreed that it should be described in the report and 1 (6%) had no opinion. Regarding the extension of the lesion to the first branch of the superior mesenteric vein, 16 (89%) agreed that it should be described in the report and 2 (11%) had no opinion.

### Evaluation of extrapancreatic involvement

All 18 participants agreed that the radiology report should describe the presence or absence of the following: hepatic lesions, whether suspected, indeterminate, or benign; peritoneal nodules; ascites; enlargement of hepatic hilar, celiac trunk, splenic, periaortic, or interaortocaval lymph nodes; and the invasion of adjacent structures.

## DISCUSSION

By having specialists in pancreatic diseases complete a questionnaire covering several aspects of the CT scan for the evaluation of patients with PDAC, it was possible to devise a model of a structured tomography report that not only meets the expectations of those professionals but also improves communication among the various specialists involved in the care of this population of patients. To our knowledge, this is the first initiative aimed at creating a Portuguese-language radiology report of this type.

A diagnosis of PDAC continues to pose a therapeutic challenge at cancer treatment centers around the world^(5,22)^. Cure is most likely to be achieved through complete surgical resection,^(18)^ and a tumor-free surgical margin is directly related to patient survival time^(22-24)^. In addition, unnecessary extension of the margin of safety during surgery does not have a significant impact on the survival of PDAC patients but can increase the morbidity associated with the procedure^(22,23)^. Staging performed by imaging methods, including CT, plays a key role in the stratification of these patients and in the choice of the appropriate therapy^(13)^. Clear, complete communication of this information to the attending physician is crucial to guaranteeing better therapeutic results for the benefit of the patient^(19)^. However, the current imaging reports for PDAC patients have many limitations, such as variability in the terms used to define the extent of the disease, as well as incomplete descriptions, which can alter the prognosis and therapeutic planning^(18)^. There are also discrepancies between the main terms used by radiologists and their interpretations by attending physicians^(25)^.

Many initiatives have been undertaken to improve the quality of radiology reports^(26,27)^. In this context, models of structured reports have been proposed for use in various clinical conditions, notably those proposed by the American College of Radiology^(26)^. However, there is still some resistance to such models among radiologists, mainly due to difficulties inherent to the implementation of structured reports, which demand more time and energy on the part of the radiologists^(20)^. Some studies have compared structured reporting and free-text dictation, in terms of their advantages and disadvantages^(19,28,29)^, without necessarily demonstrating an increase in the accuracy of the report^(28)^ or in its clarity^(29)^. However, when the impact was analyzed specifically in patients with pancreatic neoplasms, the structured report presented benefits in the therapeutic planning, due to the guaranteed inclusion of crucial aspects in the report, as well as the greater detail and clarity in the information transmitted^(19)^.

In our study, the team of specialists assembled agreed that the main aspects proposed by other pancreatic disease study groups for the evaluation of pancreatic carcinoma were fundamental for inclusion in the radiology report^(18)^. It was therefore possible to propose a model of a structured report for PDAC, validated by Brazilian professionals and adapted for use in our country (Table 3). Detailed schematic drawings illustrating the criteria for vascular invasion are freely available in the literature^(18)^.

**Table 3:** Model of a structured report for PDAC, based on the opinions of specialists working in Brazil.

**Morphology of the lesion**
Enhancement: hypovascular/isovascular/hypervascular
Size of the lesion at its greatest diameter: ______ cm
Location of the lesion in the pancreas: uncinate process/head/body/tail
Abrupt narrowing or obstruction of the pancreatic duct: yes/no
Upstream dilatation: yes/no
Parenchymal atrophy: yes/no
Obstruction of the common bile duct: yes/no
Dilatation of the biliary tract: yes/no
**Evaluation of arterial involvement**
Superior mesenteric artery: unaffected/affected
Degree of contact between the tumor and the artery: ≤ or > 180º?
Focal stenosis: yes/no
Irregular vessel contour: yes/no
Celiac trunk: unaffected/affected
Degree of contact between the tumor and the artery: ≤ or > 180º?
Focal stenosis: yes/no
Irregular vessel contour: yes/no
Common hepatic artery: unaffected/affected
Degree of contact between the tumor and the artery: ≤ or > 180º?
Focal stenosis: yes/no
Irregular vessel contour: yes/no
Extension to the bifurcation: yes/no
Anatomic variation: yes/no
Which? _______________________________: unaffected/affected
Degree of contact between the tumor and the artery: ≤ or > 180º?
Focal stenosis: yes/no
Irregular vessel contour: yes/no
**Evaluation of venous involvement**
Portal trunk: unaffected/affected
Degree of contact between the tumor and the vein: ≤ or > 180º
Focal stenosis: yes/no
Irregular vessel contour: yes/no
Venous thrombosis: yes/no
Superior mesenteric vein: unaffected/affected
Degree of contact between the tumor and the vein: ≤ or > 180º?
Focal stenosis: yes/no
Irregular vessel contour: yes/no
Venous thrombosis: yes/no
Thrombosis of the splenic vein: yes/no
Extension to the first branch of the superior mesenteric vein: yes/no
Collateral circulation: yes/no
Location: peripancreatic, mesenteric, in the hepatic hilum, or in the left hypochondrium
**Evaluation of extrapancreatic involvement**
Hepatic lesions: yes/no
Suspicious, indeterminate or benign
Peritoneal or omental nodules: yes/no
Ascites: yes/no
Suspicious or enlarged lymph nodes: yes/no
Location: hepatic hilum, celiac trunk, splenic hilum, periaortic region, or
interaortocaval region
Invasion of adjacent structures: yes/no

Our study has some limitations. Our sample was relatively small, only 18 experts participating in the study. However, it should be noted that the consensus statement issued jointly by the Society of Abdominal Radiology and the American Pancreatic Association^(18)^ was based on the opinion of only 15 experts. Because pancreatic neoplasms are less prevalent than are other diseases of the digestive system, it is difficult to assemble a great number of experts specializing in the field. In addition, our sample consisted mainly of surgeons, who accounted for 66.7% of the sample. That is understandable, because surgical resection is the treatment of choice for PDAC and is therefore the main target of radiology reports. Furthermore, the specialists consulted in this study had been trained in the city of São Paulo, where they conducted most of their professional activities. Therefore, our results do not necessarily reflect the panorama of opinions or the opinions of radiologists in the rest of Brazil. However, the respondents were affiliated with institutions that are major centers of research and medical education, their responses therefore reflecting, in part, the opinions held and practices adopted at many other referral centers.

Although structured reports are still rarely used by radiologists, they can provide major benefits in the evaluation, treatment, and follow-up of patients with PDAC, being well accepted by the professionals involved in the care of such patients, mainly because of the ease at which information essential for guiding practice can be extracted. Therefore, we have proposed a model of a structured abdominal CT report based on the opinions of experts working in Brazil. Its adoption should ultimately guarantee the transmission of information important to benefit patients with PDAC, allowing practitioners to avoid unnecessary surgical procedures and to identify patients who could effectively benefit from a treatment considered curative.

## Figures and Tables

**Figure 2:**
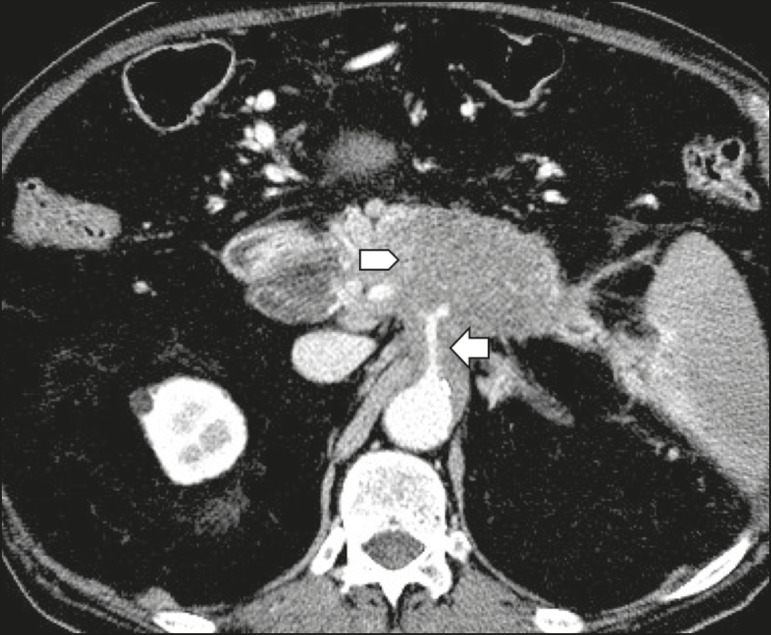
Neoplasm in the body and tail of the pancreas (arrowhead), with involvement of the celiac trunk (arrow). The mass completely envelops the vessel, up to its origin at the abdominal aorta.

**Figure 3:**
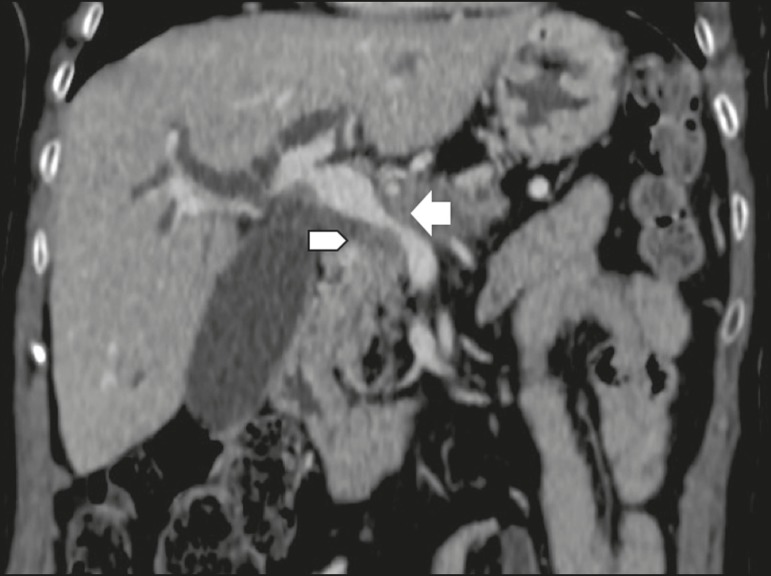
Neoplasm in the head and neck of the pancreas, with involvement of the portal vein, resulting in dilatation of the common bile duct and intrahepatic biliary tract. Pancreatic tumor (arrowhead), enveloping, narrowing, and deforming the portal vein (arrow).
